# Thrombolysis in Stroke within 30 Minutes: Results of the Acute Brain Care Intervention Study

**DOI:** 10.1371/journal.pone.0166668

**Published:** 2016-11-18

**Authors:** Sanne M. Zinkstok, Ludo F. Beenen, Jan S. Luitse, Charles B. Majoie, Paul J. Nederkoorn, Yvo B. Roos

**Affiliations:** 1 Department of Neurology, Academic Medical Center University of Amsterdam, Amsterdam, the Netherlands; 2 Department of Radiology, Academic Medical Center University of Amsterdam, Amsterdam, the Netherlands; 3 Department of Emergency Medicine, Academic Medical Center University of Amsterdam, Amsterdam, the Netherlands; Hopital Robert Debre, FRANCE

## Abstract

**Background and Purpose:**

Time is brain: benefits of intravenous thrombolysis (IVT) in ischemic stroke last for 4.5 hours but rapidly decrease as time progresses following symptom onset. The goal of the Acute Brain Care (ABC) intervention study was to reduce the door-to-needle time (DNT) to ≤30 minutes by optimizing in-hospital stroke treatment.

**Methods:**

We performed a single-centre before (pre-intervention period: 2000–2005) versus after (post-intervention period: 2006–2012) comparison in a cohort of consecutive patients treated with IVT. The intervention consisted of the implementation of a multidisciplinary stroke protocol combining simple strategies to reduce the DNT. Primary endpoint was the DNT, presented as proportion ≤30 minutes and median time. Secondary clinical endpoints were symptomatic intracranial hemorrhage (SICH), and favourable outcome defined as a modified Rankin scale (mRs) score of 0–2 at 3 months. Endpoints were additionally adjusted for baseline imbalances between the groups.

**Results:**

In the pre-intervention period, none (0.0%) of the 100 patients (mean age 63.8 years, median National Institutes of Health Stroke Scale [NIHSS] score 14) treated with IVT had a DNT ≤30 minutes compared to 234 (62.7%) of the 373 patients (mean age 66.7 years, median NIHSS score 10) in the post-intervention period (p<0.001). The median DNT decreased from 75 (IQR 60–105) to 28 minutes (IQR 20–37, p<0.001). SICH rate remained stable (3.0% versus 4.4%, OR 1.50, 95% CI 0.43─5.25; adjusted OR 5.47, 95% CI 0.69–42.12). The proportion of patients with a favourable outcome increased (38.9% versus 52.3%, OR 1.72, 95% CI 1.09–2.73) but lost statistical significance after adjustment (adjusted OR 1.46, 95% CI 0.82–2.61).

**Conclusions:**

Important and sustained reduction of the DNT to 30 minutes or less can be safely achieved by optimizing in-hospital stroke treatment. With its simple strategies, the ABC-protocol is a pragmatic framework for increasing the therapeutic yield in time-dependent stroke treatment.

## Introduction

Intravenous thrombolysis (IVT) with alteplase—either alone or followed by endovascular thrombectomy—is an effective treatment for acute ischemic stroke. [1,2] Benefits of IVT rapidly decrease as time progresses following symptom onset: the number needed to treat to recover without disabilities is 10 within the first 3 hours after symptom onset but doubles when patients are treated between 3 and 4.5 hours in a pooled analysis of randomised trials. [3] This time-dependent effectiveness has recently been confirmed in daily clinical practice. [4,5]

Guidelines recommend hospitals to achieve door-to-needle time (DNT), the time between hospital arrival and initiation of IVT of 60 minutes or less. [6,7] In daily practice, this limit is rarely met: the large observational Safe Implementation of Treatment in Stroke International Stroke Thrombolysis Registry reports a median DNT of 67 minutes with no change in DNT during the 9-years registry period. [8] Even hospitals participating in the US ‘Get With the Guidelines-Stroke’ improvement program, reported DNTs was below 60 minutes in only half of the cases. [9] Knowing that every minute a large-vessel stroke goes untreated, an estimated 1.9 million neurons are potentially lost, [10] we should be more ambitious. It has been shown that for a 30-minutes faster treatment, patients have a 8% greater odds of walking independently at discharge, and a 6% greater odds of being discharged to home instead of to an institution. [5] Therefore, the Acute Brain Care (ABC) intervention study aimed to reduce the DNT to 30 minutes or less by optimizing in-hospital stroke treatment.

## Methods

### Study design, setting and population

The ABC-study was a before (pre-intervention period: Jan 1, 2000 to Dec 31, 2005) versus after (post-intervention period: July 1, 2006 to Dec 31, 2012) comparison in a cohort, carried out in the Academic Medical Center (AMC), a university hospital affiliated with the University of Amsterdam. The AMC is a primary centre for all neurological emergencies and serves as comprehensive stroke centre in a densely populated urban area in the Netherlands. The study population consisted of all consecutive acute ischaemic stroke patients treated with IVT in the Emergency Department (ED). Patients could be referred by Emergency Medical Services (EMS), primary care physicians, other hospitals or arrived on the ED by self-referral. Patients with in-hospital strokes were excluded since the ABC-study protocol was only partially applicable since some procedures were already performed on the ward and strokes were often intervention-related, leading to a more individualized approach. Patients with complete resolution of symptoms upon arrival in the hospital, who subsequently developed a stroke after initial work-up, were considered as having an in-hospital stroke. At the start of the study in 2000, IVT was administered within 3 hours after stroke onset until publication of the European Canadian Acute Stroke Study III at the end of 2008, when treatment up to 4.5 hours was implemented. [9] Our study was conducted according to national legislations, and the medical ethics committee of the AMC permitted analysis of the anonymous patient data with waiver of informed consent.

### Intervention

The study intervention consisted of the development and implementation of a multidisciplinary ABC-protocol between Jan 1 and June 30, 2006. This protocol with 24/7 coverage combines multiple simple strategies to reduce the DNT (Table 1).

**Table 1:** The Acute Brain Care protocol: strategies to reduce the door-to-needle time.

**Pre-hospital phase**
Education of EMS staff to recognize and prioritise acute stroke
Hospital prenotification by EMS in case of suspicion of acute stroke
Collective pager warning of stroke team prior to patients’ arrival
Pre-registration of patient in hospital information system and pre-order of laboratory tests
Direct availability of a CT-room in ED
History, medication use and POC glucose measurement during ambulance transport
**In-hospital phase**
Direct transfer onto CT-table upon hospital arrival
Simultaneously EMS briefing, neurological evaluation, and blood withdrawal on CT-table
Treatment decision before laboratory results are available. POC INR if needed
Dedicated Acute Brain Care room adjacent to CT-room at the ED
Use of bed with built-in scales for exact weight determination
Predefined tables with weight-adjusted alteplase dosage

EMS, Emergency Medical Services; ED, emergency department; CT, computed tomography; POC, point of care; INR, international normalised ratio; IVT, intravenous thrombolysis.

See S1 Video for the operational ABC-protocol. Development of the protocol was guided by the observed delays in the pre-intervention period and international guidelines for trauma care and treatment in acute myocardial infarction. [11–13] In this 6 months period, no patients were included as the development process induced already a change of the prevailing procedures at that time, thereby diluting the effect of the intervention.

### Acute Brain Care protocol: current procedures

After an educational program for the EMS staff including instructions to recognize stroke symptoms using the face arm speech time (FAST) scale and emphasis on the importance of rapid treatment, the EMS dispatchers assign possible stroke patients as the highest priority level. [14] Since time can be saved when institutional preparations are made prior to arrival of the patient, on-site paramedics notify the ED when they suspect an acute stroke. Upon notification, all members of the 24/7 in-hospital stroke team are collectively warned by a unique pager sound 10 minutes before estimated arrival of the patient. The stroke team consisted of a neurology resident, supervised by a (stroke) neurologist, neurology nurse, radiology resident, supervised by a (stroke) radiologist, radiology technician, and an emergency nurse. Patients are pre-registered in the hospital information system and laboratory tests are pre-ordered. The CT-scanner in the ED is a sliding gantry multislice CT which can be easily moved back and forth between two rooms, of which one is prepared ahead of time to guarantee direct availability of the CT-scan. During transport, paramedics obtain patients’ history, medication use and perform a point-of-care (POC) glucose test. Upon arrival, the paramedics transfer the patient straight onto the CT-table. Whereas in the pre-intervention period diagnostic work-up was performed in serial steps, after the intervention vital checks, brief neurological examination including the National Institutes Health Stroke Scale (NIHSS), and blood drawing are all performed simultaneously by the stroke team on the CT-table. In case of uncertainty about use of anticoagulants, a point of care (POC) INR (Coaguchek^®^ XS, Roche, Almere, the Netherlands) measurement is performed. [15] Other laboratory results are no longer needed for the treatment decision if a patient is not known with a pre-existing illness that could affect haemostasis. After acquisition of a non-contrast cranial CT-scan, the radiologist interprets the CT-images immediately at the monitor. In the absence of contra-indications for IVT, patients are directly moved to a dedicated ABC-room adjacent to the CT-room. Here, patients are placed on a bed with built-in scales on which the patient is weighed. Previously, determination of the patients’ weight using a weight scale mat took more than 5 minutes. In the ABC-room, alteplase is prepared using predefined tables with weight-adjusted dosages. In case additional intra-arterial therapy is considered, patients are transported back to the CT-room for a CT-angiography during alteplase infusion. We have to note that since endovascular interventions have been proven effective [2] and are now standard of care, CT-Angiography is nowadays performed together with the non-contrast CT. When acute stroke is suspected in a hospitalized patient, the consulted neurology physician notifies the ED and arranges immediate transport to the CT-room. From that moment the same protocol is followed as if the patient had directly been presented to the ED.

### Data collection, definitions and endpoints

Demographics and time intervals of consecutive IVT patients were prospectively collected. The DNT was defined as time between arrival of the ambulance at the AMC and initiation of IVT, i.e. bolus administration, directly followed by continuous infusion. [16] Onset-to-treatment time (OTT) was defined as the time between symptom onset or moment last known well and initiation of IVT. In case of fluctuating symptoms time of initial symptoms was considered as onset time. Of all patients entered into the ABC-protocol in the post-intervention period, final diagnosis, main reason to renounce IVT and reason for a prolonged DNT (i.e. >30 minutes) were prospectively registered and used for feedback in regular ABC-meetings, which were held every 3 months. Additional baseline characteristics, symptomatic intracranial haemorrhage (SICH), defined as any CT-documented haemorrhage with a ≥4 points increase on the National Institutes of Health Stroke Scale, and final diagnosis of a stroke mimic were retrospectively abstracted from medical chart review for patients in the pre-intervention period, whereas these data were prospectively collected in a hospital-based stroke registry during the post-intervention period. [17] Modified Rankin scale (mRs) scores were obtained by a dedicated stroke nurse through a structured telephone interview with the patient or next of kin, hospital discharge letters when patient was referred to another hospital, consultation of general practitioner, or a combination, with a favourable outcome defined as a mRs score of 0–2. Primary endpoint was the DNT, reported as the proportion of DNT ≤30 minutes and as median time. Secondary endpoints were time intervals between symptom onset, hospital arrival, start of CT-scan and needle. Secondary clinical endpoints were SICH, functional outcome at 3 months (favourable outcome, mortality, and ordinal mRs score 0–5 among survivors), and patients with a stroke mimic. SICH, favourable outcome and mortality were analysed separately for patients treated within 180 minutes.

### Statistical analysis

Baseline characteristics were compared using student t, χ^2^ and Mann-Whitney U tests, where appropriate. Effect size on the proportions treated with a DNT of ≤30 was expressed as absolute difference between proportions. Median time intervals and ordinal mRs scores were compared between the periods using the Mann-Whitney U test. The effect on SICH and functional outcome was expressed as odds ratio’s (ORs) with 95% confidence interval (CI). These ORs were additionally adjusted for the main prognostic variables (age, sex, pre-stroke independency (baseline mRs 0–2), and baseline NIHSS) using multivariable logistic regression (adjusted OR). P-values <0.05 were considered statistically significant. All statistical analyses were performed with SPSS version 20 software (SPSS Inc., Chicago, Illinois, USA).

## Results

**Fig 1:**
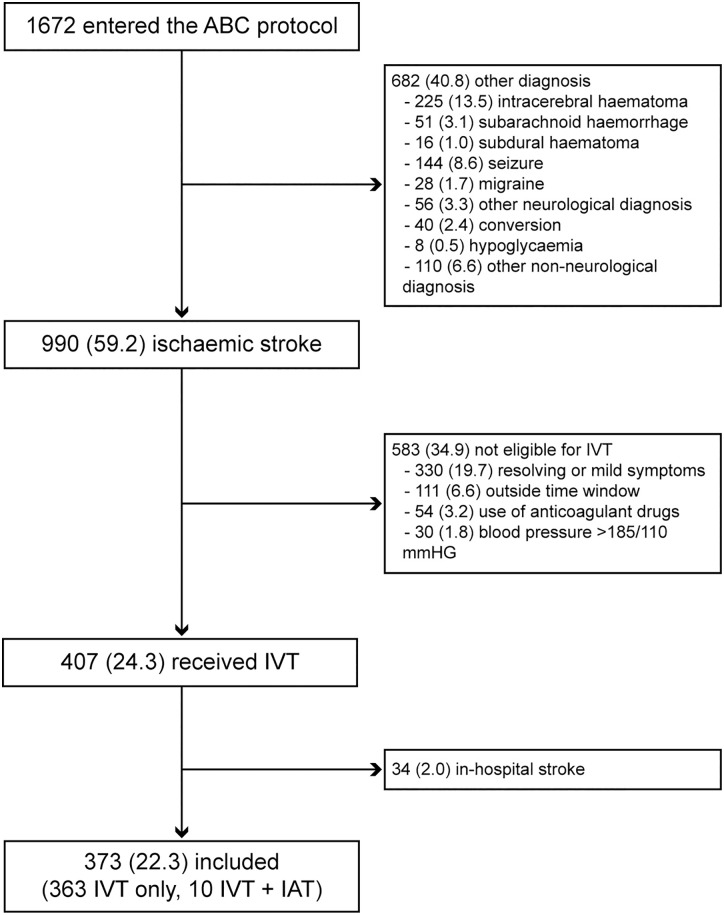
Flowchart of patients who were entered into the Acute Brain Care protocol. Legend: ABC indicates Acute Brain Care; IAT, intra-arterial therapy; IVT, intravenous thrombolysis. Ten patients who were not eligible for intravenous thrombolysis received intra-arterial therapy as primary treatment.

During the pre-intervention period 102 patients were treated with IVT, of whom 2 were excluded because of an in-hospital stroke. No endovascular interventions were performed in this phase. During the post-intervention period, 1672 patients were entered into the ABC-protocol, of whom 990 (59.2%) were diagnosed with an ischemic stroke, and another 292 patients (17.4%) suffered an intracranial hemorrhage. Finally, 407 patients (24.3%) received IVT. After exclusion of 34 patients with an in-hospital stroke, 373 patients in the post-intervention period were available for analysis (Fig 1). Of these, 10 patients received an endovascular intervention after IVT. Baseline characteristics of patients in each period are presented in Table 2. In the post-intervention period, IVT patients were more often male, were less frequent independent prior to their stroke, and had lower NIHSS scores.

**Table 2:** Baseline demographic and clinical characteristics.

	Pre-intervention (n = 100)	Post-intervention (n = 373)	P-value^a^
Age, years	63.8 (14.5)	66.7 (16.0)	0.108
Men	46 /100 (46.0)	213/373 (57.1)	0.048
Pre-stroke independency (mRs 0–2)	95/100 (96.0)	307/345 (89.0)	0.037
Hypertension	41/97 (42.3)	167/364 (45.9)	0.525
Diabetes mellitus	11/97 (11.3)	56/365 (15.3)	0.320
Hyperlipidemia	18/97 (18.6)	41/362 (11.3)	0.059
Prior ischemic stroke or TIA	16/97 (16.5)	75/363 (20.7)	0.360
Atrial fibrillation	11/97 (11.3)	39/362 (10.8)	0.874
Coronary artery disease	17/97 (17.5)	53/365 (14.5)	0.463
Current smoking	23/97 (23.7)	86/349 (24.6)	0.850
Antihypertensive drug	43/96 (44.8)	185/361 (51.2)	0.261
Antiplatelet therapy	31/97 (32.0)	128/366 (35.0)	0.578
Statin	21/97 (21.6)	92/361 (25.5)	0.437
NIHSS	14 (9 to18)	10 (5 to 17)	0.001
Systolic blood pressure, mm Hg	152 (28.2)	151 (24.5)	0.926
Diastolic blood pressure, mm Hg	84 (14.0)	83 (15.3)	0.531
Admission blood glucose, mmol/l	7.1 (2.1)	7.1 (2.2)	0.861

Data are number (%), mean (standard deviation) and median (interquartile range).

^a^ P-values were calculated using student t, χ^2^ and Mann-Whitney U tests, where appropriate.

MRs, modified Rankin scale; NIHSS, National Institutes of Health Stroke Scale; TIA, transient ischaemic attack.

**Fig 2:**
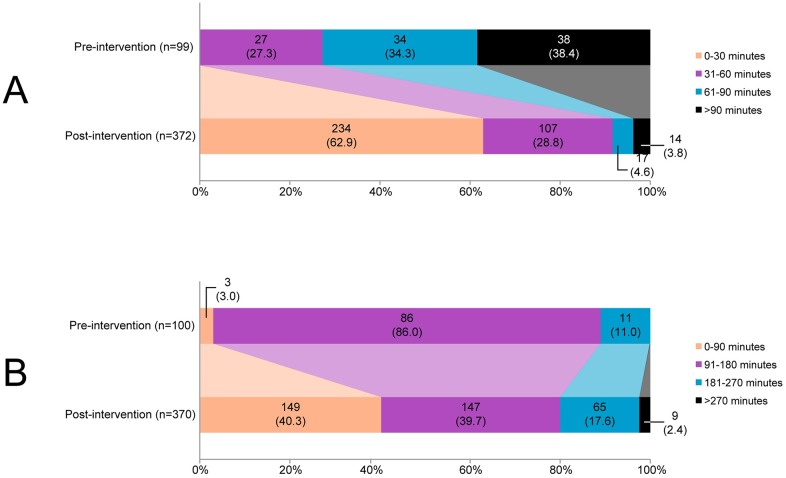
Number of patients according to the door-to-needle time (A) and onset-to-treatment time (B). Legend: Door-to-needle time was missing in 2 patients (1 in each period). Onset-to-needle time was missing in 3 patients in the post-intervention period.

The number of patients with a DNT ≤30 minutes increased from 0 (0.0%) in the pre-intervention period to 234 patients (62.7%) in the post-intervention period (p<0.001, Fig 2a). The median DTN was reduced from 75 minutes (interquartile range [IQR] 60 to 105) in the pre-intervention period to 28 minutes (IQR 20 to 37) in the post-intervention period (p<0.001). All median time intervals are presented in Table 3.

**Table 3:** Median time intervals.

	Pre-intervention (n = 100)	Post-intervention (n = 373)	P-value ^a^
Onset-to-door time	65 (50–90)	71 (48–120)	0.156
Onset-to-ambulance time	29 (12–54)	35 (18–60)	0.107
Ambulance-to-door time	38 (27–49)	32 (25–40)	0.007
Door-to-needle time	75 (60–105)	28 (20–37)	<0.001
Door-to-CT time	35 (27–47)	6 (4–10)	<0.001
CT-to-needle time	40 (31–55)	20 (15–28)	<0.001
Onset-to-treatment time	158 (135–177)	105 (75–160)	<0.001

Data are minutes (interquartile range).

^a^ P-values were calculated using Mann-Whitney U test.

Although the ambulance-to-door time slightly decreased, onset-to-door time did not change after the intervention. Reduction of both the door-to-CT-time and the CT-to-needle-time contributed to the reduced DNT, with 29 and 20 minutes, respectively. As a consequence, the median OTT was reduced from 158 minutes (IQR 135 to 177) to 105 minutes (IQR 75 to 160) after the intervention (p<0.001). Fig 2b shows a shift towards ultra-early treatment after the intervention: the number of patients treated within 90 minutes after symptom onset increased from 3 (3.0%) to 149 patients (40.3%, p<0.001).

**Fig 3:**
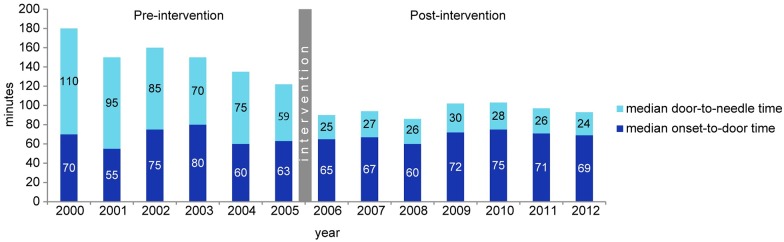
Annual median onset-to-door and door-to-needle times.

In Fig 3 the annual distribution of treatment delays is presented, showing that onset-to-door time did not vary over the years, whereas the DNT dropped down immediately after the intervention and remained stable afterwards. The annual number of patients treated with IVT increased from median 17 in the pre-intervention to 55 in the post-intervention period.

The reasons of a prolonged DNT (138 patients) in the post-intervention period is presented in Table 4.

**Table 4:** Causes of prolonged door-to-needle time in the post-intervention period.

		DNT >30 min (n = 138)
**Patient-related factors**		**68 (49.3)**
	Insufficient history or uncertain diagnosis	34 (24.6)
	Fluctuating deficits	14 (10.1)
	Uncontrolled hypertension	11 (8.0)
	Unstable patient	7 (5.1)
	Other	2 (1.4)
**Logistic factors**		**45 (32.6)**
	Self-referral of patient to ED	18 (13.0)
	Delayed or no pre-notification by EMS	6 (4.3)
	Concurrent high-priority patient	9 (6.5)
	Technical equipment problems	6 (4.3)
	Difficulties with drip insertion	6 (4.3)
**Not specified**		**25 (18.1)**

Data are number (%).

DNT, door-to-needle time; ED, Emergency Department; EMS, Emergency Medical Services.

Reasons for DNT >30 minutes were patient-related in 68 patients (49.3%). Main cause was uncertainty about the eligibility for IVT in 34 patients (24.6%), requiring additional information about the history, diagnostic studies (including CT angiography) or consultation of other physicians. The main logistic contributor to a prolonged DNT was delayed neurology consultation at the ED in 18 patients (13.0%). In these cases, patient arrived by self-referral or without prenotification of the ambulance. As these patients were no immediately recognized as a stroke at the emergency department, there was a substantial delay in the warning of the neurology physician and subsequent start of treatment. Reason for delay was not specified in 25 patients, due to relatively mild delay (DTN 35, IQR 32 to 40) in these patients.

**Fig 4:**
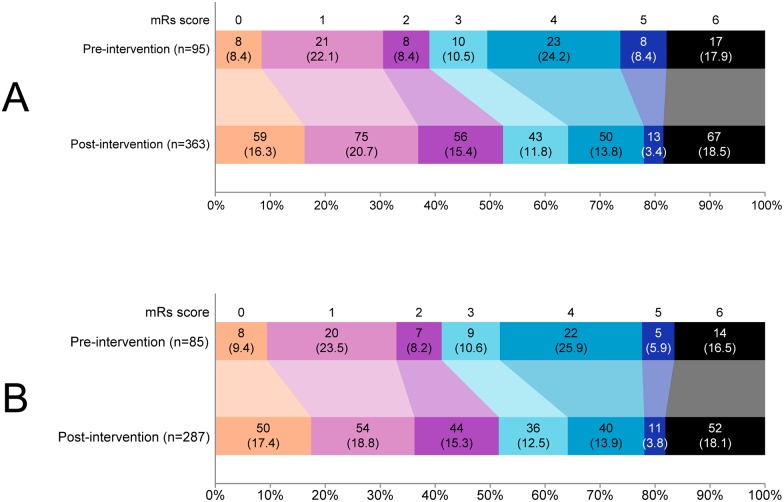
Ordinal distribution of modified Rankin scale scores at 3 months for all patients (A) and for patients treated within 180 minutes (B). Legend: mRs indicates modified Rankin scale. MRs scores were missing in 15 patients (5 pre-intervention, 10 post-intervention). Mann-Whitney U test on the mRs score 0–5 among survivors: p = 0.004; for patients treated within 180 minutes: p = 0.04.

The comparison of clinical endpoints is shown in Table 5. The number of patients with SICH remained stable: 3 (3.0%) in the pre-intervention period versus 16 (4.4%) in the post-intervention period (OR 1.50, 95% confidence interval [CI] 0.43 to 5.25, after adjustment OR 5.47, 95% CI 0.69 to 43.12). After the intervention, more patients had favourable outcome at 3 months: 37 (38.9%) versus 190 (52.3%, OR 1.72, 95% CI 1.09 to 2.73), with loss of statistical significance after adjustment (OR 1.46, 95% CI 0.82 to 2·61). Mortality at 3 months did not differ after the intervention (OR 1.02, 95% CI 0.57 to 1.84, adjusted OR 0.99, 95% CI 0.48 to 2.04). Among survivors, ordinal mRs scores improved (Fig 4a, p = 0.004). These clinical results were not altered when analysis was restricted to patients with ONT ≤180 minutes (Table 5, Fig 4b). Regarding the final diagnosis, no patients with a stroke mimic could be identified in the pre-intervention period, whereas 14 patients (3.8%) had a stroke mimic in the post-intervention period (p = 0.05). None of these stroke mimics experienced a SICH.

**Table 5:** Safety and 3-month functional outcome.

**All patients**				
	Pre-intervention (n = 100)	Post-intervention (n = 373)	OR (95% CI)	Adjusted OR^a^ (95% CI)
SICH (%)	3/100 (3.0)	16/361 (4.4)	1.50 (0.43–5.25)	5.47 (0.69–43.12)
mRs 0–2 (%)	37/95 (38.9)	190/363 (52.3)	1.72 (1.09–2.73)	1.46 (0.82–2.61)
Mortality (%)	17/95 (17.9)	67/368 (18.2)	1.02 (0.57–1.84)	0.99 (0.48–2.04)
**Patients treated within 180 minutes**				
	Pre-intervention (n = 89)	Post-intervention (n = 296)	OR (95% CI)	Adjusted OR ^a^ (95% CI)
SICH (%)	1/89 (1.1)	11/288 (3.7)	3.50 (0.46–27.45)	4.00 (0.48–33.63)
mRs 0–2 (%)	35/85 (41.2)	148/287 (51.6)	1.52 (0.93–2.48)	1.46 (0.78–2.72)
Mortality (%)	14/85 (16.5)	52/292 (17.8)	1.10 (0.58–2.10)	0.83 (0.31–2.23)

CI, confidence interval; mRs, modified Rankin scale; OR, odds ratio; SICH, symptomatic intracranial hemorrhage.

^a^ Adjusted for age, sex, pre-stroke independency, and National Institutes of Health Stroke Scale.

## Discussion

This study demonstrates that it is feasible to reduce the DNT to less than 30 minutes by optimizing in-hospital stroke treatment. This reduction was sustained given the stable DNT over the 6.5 years following the intervention. Patients were treated at earlier time points without safety concerns. The proportion of patients with a favourable outcome increased but this was largely explained by baseline imbalances between the groups. As a consequence of the focus on rapid treatment, the proportion of patients with a stroke mimic that received IVT increased, but our reported rate of 3.8% is still in the lower range of stroke mimic rates reported from other centres. [17]

The main strength of our study in a daily care setting was the comprehensive data collection that enabled in-depth analysis of the whole process and its long-term effects. We have demonstrated that 77% of all referred patients were finally diagnosed with stroke, either ischemic or hemorrhagic, which is high compared to reported rates of correct stroke identification by EMS ranging from 45 to 62% [18–21]. The high accuracy in our study can be explained by the following: training of EMS staff including use of a standardized stroke recognition protocol (FAST), the possibility of telephone consultation of the neurologist before referral, and direct feedback as patient’s evaluation mostly takes place in the presence of EMS paramedics. The reduction in DNT was largely explained by a reduction in door-to-CT time, as several actions take place at the same time, whereas in the pre-intervention period these were serial processes. In order to keep attention towards minimal delays and to identify obstacles that caused treatment delay, regular ABC-meetings are organised every 3 months. Feedback on suboptimal performance has been shown to improve the DNT. [22] For example, we observed a delay in neurology consultation in self-referred patients at the ED. When a more extended triage protocol was introduced at the ED, this problem largely diminished. This might explain the further reduction of the DNT as was observed in the last 3 years.

Several multifaceted intervention protocols aiming to reduce the in-hospital delays have been described, with reported DNTs varying between 20 and 74 minutes. [9, 23–30] Best results were obtained in Helsinki, where in the final year a median DNT of 20 minutes was achieved by “doing as much as possible before the patient has arrived while doing as little as possible after the patient has arrived at the hospital”. [26] Compared to our latest DNT of 24 minutes, this 4-minutes difference can be explained by the lack of state-wide electronic patient records in our country and no separate analysis in our study of patients with basilar artery occlusion, in whom DNT was longer. [26] In our protocol, a small delay is introduced by the weighing procedure. We felt that weighing was necessary as weight estimation is known to be highly unreliable, [31] and both alteplase underdosage and overdosage negatively affect outcome. [31,32] By using a bed with built-in scales, we follow a safe procedure with minimal delay. Additional delay is caused by our procedure to initiate IVT in the ABC-room instead of the CT-room in order to guarantee immediate alteplase infusion after bolus administration. Although introducing a delay between bolus and infusion creates time for patient transport or weighing without influencing the DNT, serum tissue plasminogen activator levels decrease when the bolus-infusion interval is longer than 5 minutes, and can consequently reduce the efficacy of IVT. [33] For correct interpretation and comparisons of reported DNT between hospitals a uniform definition of the needle-time is warranted. [16]

Our study has several limitations. First, the before-and-after design of our study cannot foresee that endpoints are influenced by external factors, like extending of the time window to 4.5 hours by the end of 2008. We therefore repeated analysis of clinical endpoints for patients treated within 3 hours after symptom onset, which showed similar results. More important was that the study population has changed over time: post-intervention patients were less often independent prior to their stroke, whilst having less severe strokes. This can be explained by a less stringent attitude towards contra-indications during the study period, as increasing experience with IVT reassured us about the safety concerns in common clinical situations. [34] Eligibility of patients with minor symptoms for IVT has been redefined recently, stating that “patients experiencing improvement of any degree, but having a persisting neurological deficit that is potentially disabling, should be treated”. [35] For these reasons, we adjusted clinical outcome for main prognostic variables, thereby losing power of our analysis. Consequently, we were not able to demonstrate that outcome has improved after intervention for the adjusted outcomes, although there is no doubt early treatment is beneficial. Secondly, we cannot compare the IVT rate between the periods as not all patients with possible acute stroke were collected in the pre-intervention period. A third point of concern is the external validity since this study was conducted in a single-centre academic setting. As patients were primarily referred by the EMS, our population does not differ from a non-academic population. Moreover, the simple strategies we implemented are not specific for our hospital, and can be reproduced elsewhere.

To summarize, important and sustained reduction of DNT to less than 30 minutes can be safely achieved by optimizing in-hospital stroke work flow and treatment. With its simple strategies, the ABC-protocol is a pragmatic framework to reduce in-hospital delays in time-dependent stroke treatment. This protocol can easily be adopted by clinicians in everyday care.

## Supporting Information

S1 Video Acute Brain Care Amsterdam. This 2-mins video movie of the operational Acute Brain Care protocol.
(M4V)
